# The Way we were: Technology will Change the Profession of Vascular Surgery

**Published:** 2020-02-20

**Authors:** A Stella

**Affiliations:** Vascular Surgery, University of Bologna, Policlinico S. Orsola-Malpighi, Bologna, Italy

## I. INTRODUCTION

In 1831 Jacob Bigelow, a Harward professor, used the word in the title of his book Elements of Technology on Application of the Sciences to the Useful Arts. He used “technology and the “pratical arts” almost interchangeably but distinguished them by associating technology with the application of science to the pratical, or useful arts. For Bigelow, the sciences consisted of discovered principles, ones that exist independently of humans. The sciences are discovered not invented. We also see technology as a creative process involving human ingenuity. The word Technology was infrequently used until the late twentieth century. When a group of about twenty American historians and social scientists formed the “Society for the Hystory of technology in 1958, they debated whether the society should be known by the familiar word “engineering” or the unfamiliar one “Technology”. They decided upon the latter, believing “technology”, though the less used and less well defined term, to be a more inclusive term than “engineering”, an activity that it subsumes. So the historians of technology today are applying the word to word to activities and things in the past not then known as technology, but that are similar to activities and things in the present that are called technology. For example, machines in the nineteenth century and mills in the medieval period are called technology today, but they were not so designed by contemporaries, who called them simply machines and mills. Technology, creativity and innovation are terms used in the same process describing different aspects of the human activities 1.

The term of innovation derives from the Latin word “innovatus”, which is the noun form of “innovare” means to renew or change, comes from in-“into” + novus-“new”. Although the term is broadly used, innovation generally refers to the creation of better or more effective products, processes, technologies, or ideas that are accepted by markets, governments, and society.

During the development of our discipline many men and many discoveries have changed the way we see cardiovascular diseases and also the way we treat them. Innovation and technology have been the impetus for the modernization and the profound change of our profession.

## II. THE WAY WE WERE

### Prosthetic Graft

After the Aortic Transplantation made by Charles Dubost in Europe, due to the efforts of Arthur Voorhees, Sterling Edwards, and Michael DeBakey, prosthetic graft replacement for aortic aneurysms had become a reality by the mid-1950s. Many forefathers of vascular surgery were involved in the innovative and disruptive technology of graft development, but Michael DeBakey and his wife’s sewing machine were the genesis of the Dacron graft. It is a fascinating story, which could not be relived today in our hyper-regulatory environment. As retold by Dr DeBakey in a Journal of Vascular Surgery interview in 1996, he was interested in purchasing nylon cloth material to make a vascular graft as had been suggested by Voorhees. He went to the department store; they were out of nylon but did have this new cloth material, Dacron. He bought some sheets of Dacron and using his wife’s sewing machine created Dacron tubes. A couple of years of laboratory work that involved replacing the aorta of dogs with the new Dacron graft convinced him it was time to move the concept of Dacron graft replacement to patients. So he did, and the rest is history^2^. M DeBakey and Stanley Crawford pioneered much of aortic surgery, demonstrating that large sections of the aorta including those with visceral/renal branches could be replaced with success.

There are many other important surgeons in the development of aortic surgery, but we have to remember Emerick Szilagyi who documented that aortic replacement for abdominal aortic aneurysms was safe and more effective than nonoperative management, well accepted today as a given, although the threshold for repair continues to be a subject of debate in the era of endovascular aneurysm repair (EVAR). Until EVAR, open replacement of the aorta by a Dacron graft was the procedure of choice for treatment of an abdominal aortic aneurysm. Few thought this would ever change, and it would not have without the advent of endovascular therapy.

In the 1950s, the concept of approaching vascular disease through the artery as opposed to outside the artery was yet to be born. Vascular disease was approached from outside in, with either replacement of the diseased vessel or removal of the disease by endarterectomy, a technique developed by Dos Santos and popularized for carotid and many other surgeons world wide, The used technology was represented by hands, eyes, motor skills, and surgical judgment in the operating room to fix the problem 2.

### Endovascular Technology

Charles Dotter, a radiologist at University of Oregon described and performed the first endovascular intervention trying to treat vascular disease from inside the blood vessel without the large incision and minimize patient discomfort and accelerate recovery. As he stated in a 1963 address at the Czechoslovakia Radiologic Congress, “The angiographic catheter can be more than a tool for passive means for diagnostic observation, used with imagination it can become an important surgical instrument”: he was clearly way ahead of his time, considered by many of his peers and all surgeons as “Crazy Charlie”.

He detailed the procedure of transluminal angioplasty in a 1964 publication in Circulation, a paper that should be in every vascular surgeon’s library. His paper described “transluminal angioplasty” of arterial stenoses and occlusions using graduated Teflon dilators that were passed endoluminally under fluoroscopic guidance. The concept of “Dottering” the lesion was born and with the later balloon angioplasty angioplasty by Dr Andreas Gruentzig and the metal endoluminal stent by Dr Julio Palmaz, effective catheter-based therapies were established. But our reticence to adopt catheter-based therapies was significant 2.

### By Aortic Endovascular Repair to Endovascular Era

In the year 2000, cardiologist and radiologist realized much earlier the role of endovascular procedures in the treatment of cardiovascular diseases. We were destined to live the legacy of our colleagues in cardiac surgery, and it is quite possible vascular surgery would have missed the endovascular train leaving the station if it was not for Juan Parodi 1.

He presented his initial experience at a SVS national vascular meeting in the early 1990s. A case in point was his seminal article, which described his unique innovation. The manuscript was initially rejected for publication by a major vascular journal with an editorial comment to Dr Parodi that it was a crazy idea 8, but finally, the article was published in the Annals of Vascular Surgery. Fast forward to today, reflect for a moment on the consequence of his innovation.

It has resulted in all the benefits first advanced by Dotter but not imagined by Dotter for patients with aortic disease. Furthermore, it was Parodi’s innovation more than any other collegues that made it impossible for the house of vascular surgery.

But application of Parodi’s intervention require technology for performing and interpreting imaging of the vascular system, and so our konwledge changed creating competency and requirement of every vascular surgeon in practice today.

After early years of EVAR practics, Aortic Complex procedures are now possible using the sophisticated Computed Tomography (CT) and Magnetic Resonance (MR) imaging available in current practice. These technologies developed in the 1980s have permitted the successful application and expansion of Parodi’s idea. Without body imaging technology, successful endovascular therapy of the aorta is less feasible or may be not feasible at all.

Palpation of pulses, auscultation with a stethoscope, and static angiographic images were thought to be all that was required to treat a vascular patient.

The development of “noninvasive imaging” technology was fundamental to defining our specialty developing in to the endovascular treatment of peripheral arterial disease. In the 1970s Eugene Strandness, for many of Vascular Surgeons the putative “Father of Noninvasive Vascular Diagnostics” Professor of Surgery at the University of Washington with a team of engineers published in 1974 the first paper describing the synthesis of B-mode imaging with Doppler flow detection 3.

Inherent in this innovation was the concept of vascular imaging combined with the objective physiologic measurement of blood flow.

Through this technology, we have been able to monitor and correct vascular disease before the clinical event and it has revolutionized our approach as surgeons to the longitudinal follow-up of our patients, which is a unique element of our specialty.

## III. THE TECHNOLOGY WILL CHANGE THE PROFESSION OF VASCULAR SURGEONS

Veith in 2016 described well the scenario of Vascular Surgery after the entry of technology into our profession “Following the use of these technologies, already 90% of the aortic pathology, 70% of the PAD and 40–50% of carotid stenoses can be treated with endovascular techniques”. Among the different fields of action of the vascular surgeon I believe that the treatment of Thoracic Abdominal Aneurysms and shortly after the aortic arch represent this change well, showing all of us how technology makes us make epochal changes 25.

Thoraco-abdominal aortic aneurysms (TAAAs) considered challenging clinical scenarios are today the field of a big change of skills used by surgeons. Despite modern intra/peri-operative adjuncts, TAAA open repair (OR) has not negligible mortality and morbidity rates also in high volume centres. Coselli et al 4 reported the largest experience of TAAAs treated by OR (3309 cases) with 8% of operative death, 5% of spinal cord ischemia and 6% of post-operative dialysis. Post-operative cardiac and pulmonary complications were 26% and 36%, respectively1. Results may be worse in centers with lower case volume / experience.

Fenestrated and branched endografts (FB-EVAR) are nowadays available therapeutic options to treat aortic aneurysms involving renal and splanchnic arteries. Several experiences reported the FB-EVAR feasibility and effectiveness for juxta/para-renal abdominal aortic aneurysms 5–7.

For the first time, in 2001 Chuter et al 8 reported the total endovascular repair of TAAA by branched endograft. In 2010, Bakoyiannis et al 6 reported the first literature review of the TAAAs endovascular repair by FB-EVAR. Only 7 studies (with early results) were collected for an overall of 155 cases. Technical success was 94%, 30-day was 7.1% and spinal cord ischemia was 9.6% (paraparesis 7.1%, paraplegia 2.5%)6. Re-interventions occurred in 17% 9.

In the last years, thanks to the technology evolution and the increased operators’ experiences, several papers were reported with encouraging early and mid-term results. Table 1 and 2 summarize peri-operative and follow-up results of papers published in the last years with more than 150 cases 10–14.

## IV. RESULTS

Between January 2010 and July 2017, an overall of 80 TAAAs (urgent and elective cases) were treated in our centre by FB-EVAR. The mean age was 72.5 - SD 7.0 years and the mean TAAAs diameter was 63.0 - SD 18.3mm. As regards Crawford’s Classification 1.3% were type 1, 23.8% type 2, 27.5% type 3 and 47.4% type 4. Five cases were Ruptured TAAAs (6.3%). Custom made and off the shelf device were used in 57 (71.2%) and 23 (28.8%) cases for an overall of 289 target visceral vessels (3.6 / patient). Technical Success was 92.5%. Technical Failures consisted in 1 Iliac Rupture (1.3%) and 5 Renal Arteries Loss (6.2%). The post-operative target visceral vessels patency rate was 97.0%. Spinal cord ischemia was 6%. Cardiac and Pulmonary Complications rates were 6.3% and 7.5%, respectively. A significant post-operative renal function worsening (>30% of baseline) occurred in 13.8% with a new onset of dialysis of 1.3%. The 30-day mortality was 3.8%. The mean follow-up was 18.5-SD 15.0 months. The overall survival was 96%, 83%, and 70% at 6, 12 and 24 months. The overall target visceral vessels (TVV)-patency was 95%, 93%, and 93% at 6, 12 and 24 months, respectively. The overall freedom from re-interventions (FFR) was 87%, 86% and 86% at 6, 12 and 24 months, respectively. These results are comparable with the current literature data published by high volume centers for FB-EVAR TAAAs procedures 10–14.

## V. DISCUSSION

In our opinion the advantages of new technologies, that have allowed to improve results of FB-EVAR for TAAAs in the last years, can be summarized in the following points: Pre-operative Planning tools, Hybrid Rooms & vessels navigator, Staged procedure, Off the shelf endograft, Learning Curve/ Dedicated team/ High Volume.

Preoperative planning is a crucial moment of a successful FB-EVAR procedure. Case planning for FB-EVAR requires expertise in computed tomography angiography analysis and the ability to design a multi-modular endograft with fenestrations or branches according to the aorto-iliac anatomy. It is time consuming and requires experienced team and instruments. Dedicated software are nowadays available in order to perform an accurate aorto-iliac and visceral vessels anatomical evaluation. By computed tomography post processing software, we can create volume rendering, multi planar and centre lumen line reconstructions or angiographic simulation. According with these reconstructions, particular evaluations con be performed in order to plan a custom made endograft, the endovascular strategy and to optimize the entire procedure (patient position, access and amount of contrast media).

Hybrid rooms, combining open surgical environment and advanced imaging capabilities are currently replacing mobile C-arms in the operating room 15. The latest hybrid rooms have advanced imaging applications, such as contrast-enhanced cone beam computed tomography and preoperative computed tomography angiography (CTA) image fusion 15. The latter facilitates endovascular navigation (vessel navigator – a sort of 3D road map) and increases the accuracy of endograft implantation and the target visceral vessels cannulation 12. Literature data demonstrated that hybrid room and vessels navigator significantly reduce the exposure to radiation (for both patients and physicians) and the total amount of iodinated contrast injection during FB-EVAR12.

Spinal cord ischemia remains a catastrophic complication after the TAAA repair. After OR, the rate of SCI ranges between 4 and 11% 4,16,17 and it is related with the extension of the aortic disease (TAAA type II > I > III e > IV) 4,16–17. After the endovascular repair of TAAA, the SCI ranges between 3 and 17% 12 if we considered the first experiences.

Recently, different endovascular/surgical strategies were proposed to reduce the rate of SCI. Kasprzak et al 19 reported a reduction at 5% of SCI by using the temporary sac perfusion branches. Maurel et al 4 associated the concept of the temporary sac perfusion with the early lower limb/pelvic perfusion (removing as soon as possible the large sheets from the femoral arteries). According to this protocol, the SCI rate was < al 3%. Both these approaches are based on the pre-conditioned theory of the spinal vascular blood supply 20–21. We always used this approach when it was possible; we always maintained the patency of both hypogastric/subclavian arteries, and we reported an overall rate of spinal cord ischemia of 6% (considering both elective and urgent cases). It is important to underline also that it is crucial the role of the anesthesiological team during the procedure and in the peri-operative period to reduce the risk of SCI (haemodynamic stability and spinal cord pressure).

Customization of an appropriate commercially available FB-EVAR design requires usually 6–8 weeks and limits a wide application of this technology in urgent patients, such as cases with large asymptomatic and symptomatic/ruptured TAAAs. In order to expand the availability of FB-EVAR technology to acute setting, “off-the-shelf” solutions have been proposed to accommodate as many different anatomical configurations as possible 22–23. Based on this platform, the first off-the-shelf 4-branched endograft, the Zenith T-Branch endograft (Cook Medical, Bloomington, IN, USA), was employed and commercially available, starting in September 2012 to treat acute TAAA. Preliminary experiences suggested that T-branch is a safe and effective therapeutics option for urgent total endovascular TAAA repair, in which a custom-made endograft is not obtainable in the due time 22,23. Recently, we reported our experience on urgent TAAA endovascular repair by T-branch with encouraging results at early and midterm follow-up 23.

In conclusion, the total endovascular TAAA repair is technical demanding, time-consuming, and requires dedicated and advanced knowledge in the endovascular materials, technologies and dedicated team for planning, procedure and peri-operative management. The expertise is a key factor to treat challenging FB-EVAR cases. In 2016 Starnes published an experience about the importance of the learning curve in these advanced procedures 24. During the course of 136 consecutive single-surgeon FEVAR implantations, the authors have demonstrated statistically significant and clinically meaningful improvements in several outcomes during the study period, including perioperative death or major complications, length of procedure, and fluoroscopy time 24. (decrease in the proportion of patients suffering perioperative death or major complications from 23.5% in the first quartile to 8.8% in the fourth quartile. After adjustment for potential confounding factors, the odds of death or major complication were cut by 52.4% per quartile increase 24.

## VI. CONCLUSION

Our discipline after having spent years to affirm its identity and autonomy with respect to general surgery, is today actually included in the specializations that take care of cardio-thoracic vascular diseases, sharing diagnostic and therapeutic pathways. The surgical instruments used are largely different, and as recommended by Dotter’s first experience, catheters, guides and metal stents have become the new surgical instruments. But the most important aspect concerns the overbearing entry into our profession of technological innovations that are decisively changing our work. The current vascular surgeon is in need of expertise in the use of technologies that were previously a prerogative of Radiologists and / or Cardiologists, CT and MR, 3D Virtual Reality simulations are close to entering our daily work. At the same time, the open surgical operations performed by our young people in training are reduced and for some of us insufficient to guarantee the necessary capacity for carrying out major interventions.

It is time to rethink the contents and training paths of Vascular Surgery because today our young surgeons are not always able to find the contents necessary to create the required skills in the field of radiology and cardiology within the current training paths. In the future, we will have to design a new territorial distribution of the Vascular Surgery Units with different surgical skills as required by the current specialist, technological and economic needs.

What about the future for vascular surgery in 10 years and beyond? What changes should we expect? First, we can share what was reported by F Veith, which with the development of technology and the increased capabilities of vascular surgeons, “By 2026, one can predict that 75% to 95% of all vascular lesions requiring treatment will undergo an endovascular procedure” 25.

How should vascular surgery deal with the decreasing numbers of complex open procedures and who should do them? One solution is to have centers to which these patients are sent and in which vascular surgeons seeking this skill can get adequate open training.

Nevertheless, Vascular surgery has progressed over time thanks to the development of other sciences such as pharmacology for heparin, engineering for imaging, we can therefore predict that the definition of the best medical therapies together with the genetic therapies will allow us to reduce the development of diseases such as atherosclerosis.

Statin drugs are just the beginning and have already decreased rates of heart attacks, strokes, and death. Better use of these drugs will make them more effective, improve our treatments, and help our patients have longer and better lives. Despite this and the possibility of even more effective lipid-lowering therapy, complications of atherosclerosis will still occur and require our interventions, probably in increasing numbers as our population ages. This ensures a continuing need for the services vascular surgeons provide^25^.

Vascular surgery has evolved more and is now further differentiated, from its general surgery ancestor, we have become more endovascular, while general surgery has become more laparoscopic. So vascular surgery is even more qualified now for separate specialty status than it was in 2007 after Europe and more recently also in USA. on the world 25–26.

Similarly, the already promising results of AAA, Abdominal aortic aneurysm. drug-coated balloons will be enhanced. All these devices are complicated with many variable factors. The bottom line is that intimal hyperplasia will be overcome by anti-proliferative drugs in all vascular beds once the best way of getting the best drug to the proper location is found. And finally, computer-enabled remote monitoring of flows within grafts and stents will allow corrective treatment before occlusion occurs. Miniaturized piezoelectric sensors are one way to do this.

Finally, we live in an imperfect world and yes, we see it in our health care system, in which doctors are able to perform unnecessary procedures for financial gain: the overtreratment is ever possible, sometime influencing the published data.

## Figures and Tables

**Table 1:** Literature experiences (>150 cases) reported in the last years by high volume centres for FB-EVAR repair. Peri-operative results

Author	Patients (n)	Technical Success (%)	30-dav Mortality (%)	Spinal Cord Ischemia (%)
Greenberg 7	406	-	5.7	4.3
Maurel 8	204	92.6	6.9	3.9
Verhoeven 9	166	95.0	8.0	9.0
Eagleton 10	354	94.0	4.8	8.8
Oderich 11	185	94.0	4.3	3.0

**Table 2:** Literature experiences (>150 cases) reported in the last years by high volume centres for FB-EVAR repair. Follow-up results

Author	Months of Follow-up (mean ± SD)	Survival (% at 24-month)	TTV – patency (% at 24-month)	FFR (% at 24-month
Greenberg 7	-	-	-	-
Maurel 8	-	-	-	-
Verhoeven 9	29 ± 19	78	97	98
Eagleton 10	23 ± 19	68	92*–98–97	64
Oderich 11	21 ± 20	68±5/72±6**	95	62

* 92–98–97: percentages referred to renal artery, superior mesenteric artery and celiac trunk, respectively.

** 68±5/72±6: percentages referred to type I–III and type IV TAAAs, respectively
